# Neurofeedback for the treatment of children and adolescents with ADHD: a randomized and controlled clinical trial using parental reports

**DOI:** 10.1186/1471-244X-12-107

**Published:** 2012-08-10

**Authors:** Nezla S Duric, Jørg Assmus, Doris Gundersen, Irene B Elgen

**Affiliations:** 1 Department of Child and Adolescent Psychiatry, Helse Fonna Haugesund Hospital, Vinjesgate 10, Haugesund 5501, Norway; 2 Center for Child and Adolescent Mental Health, University of Bergen, Bergen, Norway; 3 Center for Clinical Research, Haukeland University Hospital, Bergen, Norway; 4 Department of Research, Helse Fonna Haugesund Hospital, Haugesund, Norway; 5 Department of Child and Adolescent Psychiatry, Haukeland University Hospital, Haukeland, Norway; 6 Department of Clinical Medicine, University of Bergen, Bergen, Norway

**Keywords:** Attention-deficit/hyperactivity disorder (ADHD), Neurofeedback, Barkley rating scale for parents

## Abstract

**Background:**

A randomized and controlled clinical study was performed to evaluate the use of neurofeedback (NF) to treat attention-deficit/hyperactivity disorder (ADHD) in children and adolescents.

**Methods:**

The ADHD population was selected from an outpatient clinic for Child and Adolescent Mental Health in Norway. Ninety-one of the 275 children and adolescents ranging in age from 6 to 18 years (10.5 years) participated in 30 sessions of an intensive NF program. The reinforcement contingency was based on the subjects’ production of cortical beta1 activity (15–18 Hz). The ADHD participants were randomized into three groups, with 30 in the NF group, 31 controls in a group that was given methylphenidate, and 30 in a group that received NF and methylphenidate. ADHD core symptoms were reported by parents using the parent form of the Clinician’s Manual for Assessment by Russell A. Barkley.

**Results:**

Ninety-one children and adolescents were effectively randomized by age, sex, intelligence and distribution of ADHD core symptoms. The parents reported significant effects of the treatments, but no significant differences between the treatment groups were observed.

**Conclusions:**

NF was as effective as methylphenidate at treating the attentional and hyperactivity symptoms of ADHD, based on parental reports.

**Trial registration:**

Current Controlled Trials NCT01252446

## Background

Attention-deficit/hyperactivity disorder (ADHD) is a developmental mental disorder characterized by persistent symptoms of inattention or inattention in combination with hyperactivity and impulsivity. Its prevalence may be up to one in four [1,2]. The severity of these symptoms and its enduring nature are known to impair a person’s capacity to effectively function. Treatment programs using behavioral and pharmacotherapeutic approaches are well established [3,4]. The Multimodal Treatment study (MTA) of ADHD identified advantages of multimodal treatment [1,5,6]. Because of rapid improvements in attention and reduced hyperactivity, many children with ADHD have been treated with stimulants [7-9]. However, improvements in social and academic skills following stimulant treatment have not been reported [9]. In addition, concerns with the benefits and side effects of long-term stimulant treatment have also been noted [10,11].

Many clinical trials have found that neurofeedback (NF) effectively treats the symptoms of ADHD [12,13]. The effects of NF have been described as improved attention, decreased hyperactivity, and increased academic and social skills [1,14,15]. However, other studies have only found improvements in attention [16], and two studies with large sample sizes did not find significant improvements in core ADHD symptoms [11,17].

NF has been discussed as being an effective ADHD treatment when given alone or in combination with medications [11]. One study did not find significant treatment responses between Ritalin and NF [17].

NF is a learning process in which the brain is rewarded for positive changes in its activity [13]. The response to this learning process is visual or auditory feedback. In NF, the placement of the electrodes and the frequency of stimulation are important. The International 10-20 system is a recognized method to describe and apply the location of scalp electrodes in the context of an electroencephalography (EEG) exam or experiment. Most NF studies have used the standardized electrode placements Cz, C3, and C4 [14]. Some studies included frontal electrodes when using NF to treat ADHD, such as the Fz, F3, and F4 electrodes [14]. Several NF protocols for treating ADHD are available. Single-channel protocols (unipolar) developed by Lubar and interhemispheric (bipolar) protocols developed by Othmers are widely practiced and supported by large-scale clinical studies [18-20].

Different approaches regarding NF treatment have been developed for ADHD [1,12,21]. The most frequently used frequencies enhance beta (15-18 Hz) and inhibit theta (4-7 Hz) brain activity [22]. Sensorimotor rhythm (SMR; 12-15 Hz) protocols that enhance SMR activity (low beta activity), alpha/theta (8–11 Hz/4–7 Hz) protocols that enhance alpha brain activity, and SCP (slow cortical potentials) protocols that reward polarity changes in EEGs are also used [23]. Usually no more than two different treatment protocols are used in NF treatments [22].

The lack of large and controlled studies may have limited the use of NF treatments in the clinic [24-27]. Many prospective controlled studies have used either stimulants or waiting lists as the control groups [17]. Only a few studies were randomized and controlled trials [16,17,28-30].

In addition, several non-randomized studies found a large effect size (ES) for attention and a medium ES for hyperactivity [31], but a randomized study by Arns and Linden found a small ES for hyperactivity [14,32].

Use of sham feedback (placebo) for evaluating the efficacy of NF in the ADHD population was declared unethical by the University of California, San Diego [24,33]. This is the most likely reason why standard medication treatments have been applied to the control groups in NF research studies.

The present study was a controlled and randomized clinical study that included children and adolescents with ADHD, who were followed at a Child and Adolescent Mental Health Clinic in Norway. The aim of the study was to evaluate the effects of NF on the core symptoms of ADHD, including attention and hyperactivity.

## Methods

### Subjects

**Figure 1:**
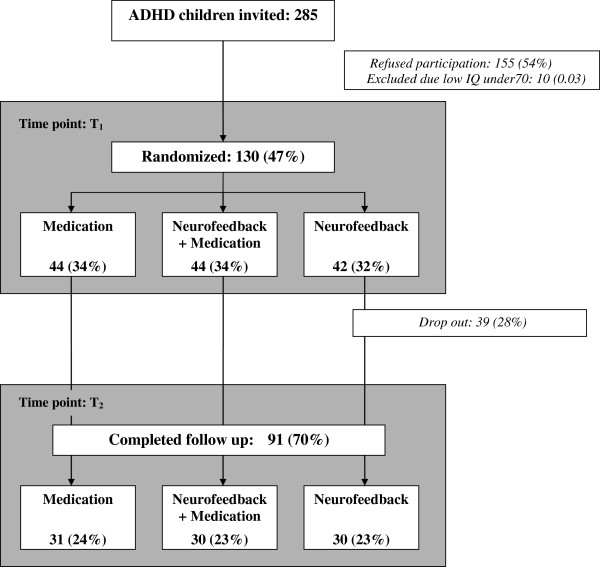
Clinical population of children with ADHD who were invited to participate in this randomized neurofeedback trial.

Over a period of 3 years (2007-9), 628 children and adolescents aged six through 18 years were referred for the treatment of ADHD to the Child and Adolescent Mental Health Clinic, Haugesund Hospital, Rogaland County in Norway. Of these, 285 (45 %) met the criteria for ADHD according to the International Classification of Disease (ICD-10) (Figure 1). All children and adolescents with ADHD were invited to participate in the study. Ten of these subjects were excluded because of a low IQ (<70), and 155 refused to participate. These children were of a similar gender and age as the participants. After randomization, 39 children and adolescent dropped out of the study 13 randomized to NF group, 15 to medication group and 11 belonged to combined NF/medication group. The treatment was completed successfully in 91 (70 %) children and adolescents (Figure 1).

### ADHD

The ADHD-referred population through 1 year (2007) has been described in detail in earlier papers [34,35]. Children referred for ADHD underwent a clinical assessment. The assessment included a child appropriate medical examination, a clinical psychiatric interview, and observations to assess ADHD and other appropriate diagnoses. Questionnaires regarding ADHD were filled in by the child, or by the parent or teacher of the child. The medical examination was done to exclude somatic conditions as the cause of the ADHD symptoms. A child psychiatrist evaluated the assessments and categorized the child as having ADHD or a non-ADHD condition according to ICD-10 diagnostic criteria.

### Inclusion criteria

All children with ADHD met the following criteria to be included in the study: 1) symptomatology consistent with ICD10 criteria for the diagnosis of ADHD; 2) age 6–18 years; 3) cognitive function above IQ 70*.* The children were evaluated using the Wechsler Intelligence Scale for Children (WISC-IV) [36,37].

### Treatment groups

The children with ADHD were randomly placed into three groups: 1) the NF group received NF; 2) the NF + MED group received NF and methylphenidate (MPH); and 3) the MED group received MPH (Figure 1).

### NF

NF is designed to change certain types of EEG activity [38,39]. The goal of the NF treatment was to enhance beta and depress theta activity [19].

During the NF treatment, the patient received feedback regarding their own brain activity. Unipolar placed sensors on the scalp measured brain activity, and a computer processed the signals as brainwave frequencies. The flow of this activity is shown to the subject, who attempts to change the activity level. Some frequencies are susceptible to promotion and others are susceptible to being diminished. Such information was presented to the children and adolescents in the form of a video game or film. When the children and adolescents played the video game or watched the films, they produced brainwave activity that was "shaped" toward more regulated performance. NF in the study was conducted using the Infinity software and equipment supplied by the biofeedback/NF and psycho-physiological instrument manufacturer Thought Technology [40].

A beta/theta NF treatment protocol for ADHD was used, including the appropriate frequency ranges for the EEGs and electrode placements. Beta enhancement (16–20 HZ) and Theta suppression (4–7 Hz) were assessed. The treatment site used was in the central area and unipolar electrodes was placed on the Cz, whereas the ground electrode was placed on the earlobe [18].

NF was conducted three times a week with 30 treatments for each child and adolescent. Each treatment lasted 40 minutes and was separated into pre and post 5-minute baseline periods (alpha training) and 30 minutes of NF (beta/theta training). Theta activity was defined as 4–7 Hz, alpha activity as 8–12 Hz, SMR activity as 13–15 Hz, beta activity as 16–20 Hz, and electromyography (EMG) activity as 80–150 Hz activity [41]. The events above these threshold levels were monitored. The standards used in the treatment were to decrease theta activity by inhibiting high amplitude theta activity or by rewarding high amplitude beta activity. The treatment effect was defined as increased beta/SMR activity of 13–20 Hz, decreased theta activity of 4–7 Hz, and decreased EMG activity.

Subjects in the MED and NF/MED group were administered methylphenidate (MPH) twice per day at the recommended dose 1 mg per kg with the final medication doses from 20 to 60 mg daily doses.

### Pre- and post-evaluation

Two core symptoms of ADHD were evaluated. Attention and hyperactivity were evaluated using the Clinician’s Manual for the Assessment of Disruptive Behavior Disorders – Rating Scale for Parents, from Russell A. Barkley [42]. The manual offers an effective tool for assessing attention and hyperactivity. The scale is divided into subscales for inattention and hyperactivity, and a total score. Normative data are rated according to the age (5–18 years) and gender of the subject. The children were evaluated in two different periods during the study (T_1_, T_2_). The baseline evaluation T_1_ was completed prior to the treatment. The effects of the treatments were evaluated as a change in ADHD core symptoms from T_1_ to T_2_. All children underwent post-treatment evaluations approximately 1 week after the NF had been completed (T_2_).

The Regional Ethics Committee on Medical Research approved the project protocol used in this study, and written consent was obtained from all subjects and their parents.

### Statistical Analysis

Basic descriptive methods (descriptive, Analyses of Variance (ANOVA), exact *χ*^2^ test) were used to investigate the baseline data. The treatment effects were investigated using a general linear model (GLM) for repeated measures, which was implemented for each of the subscales (total score, inattention, and hyperactivity). The model included the raw scores at both time points as dependent variables and the treatment groups as factors. We fitted both an unadjusted model and a model adjusted for sex and age. Even if the adjusted model may be more complete and more reliable, it was reasonable to examine the unadjusted model because of the small sample size and concomitant lack of power in the adjusted model. In the GLM, we estimated both within-subjects contrasts to test treatment-induced changes in the ratings and between-groups effects to test for differences between the treatments. Significant differences were further examined using post-hoc tests. Additionally, we estimated the standardized ES δ_RM_ for each treatment change [43].

Note that we used a negative definition of the treatment changes (T_1_-T_2_) because we wanted an improvement associated with a positive number.

The general significance level was set to 0.05. For the baseline investigation we had to take into account the effects of multiple comparisons. Because of the high correlation between a number of the investigated variables (Barclay ratings), a Bonferroni corrections method would be too conservative. As such, we decided to set the significance level to 0.01, as a compromise between a Bonferroni correction and not accounting for multiple comparisons. In the GLM, we investigated only highly correlated variables, thereby reducing the number of comparisons. Therefore, we did not adjust the significance level. All computations were done using PASW 18.

## Results

### Subjects and randomization

There were no significant differences in demographic factors or ADHD core symptoms between the treatment groups at baseline (T1) (Table 1).

**Table 1:** A clinical randomized controlled Neurofeedback study with 91 participants

				**Neurofeedback +**	
	**Total**	**Medication**	** Medication**	**Neurofeedback**	
	**Mean (SD)**	**Mean (SD)**	**Mean (SD)**	**Mean (SD)**	**p-value**
*Demografics*					
Children participating^1^	91 (100 %)	31 (34 %)	30 (33 %)	30 (33 %)	
Sex (boys)^1^	72 (80 %)	27 (87 %)	23 (77 %)	22 (73 %)	0.51^4^
Age	11.2 (2.8)	10.9 (2.4)	11.2 (2.8)	11.4 (3.1)	0.77^2^
IQ	87 (14)	87 (15)	85 (13)	89 (14)	0.75^2^
*ADHD core symptoms*^*3*^					
Total	34.1 (8.9)	35.8 (10.8)	33.8 (7.8)	32.6 (7.7)	0.39^2^
Attention	16.7 (4.7)	17.4 (5.1)	17.2 (4.9)	15.5 (4.0)	0.22^2^
Hyperactivity	17.3 (5.2)	18.2 (6.2)	16.5 (4.9)	17.1 (4.5)	0.47^2^

^1^ Number (%).^2^ ANOVA for the null-hypotheses that the values for the randomized groups are equal. ^3^ Barkley rating scale based on parents score. ^4^ Exact χ^2−^–test.

Almost one third of the subjects dropped out of the study (Figure 1). Most of them did not start the treatment because of their parents’ decision (30/39) or because of the subject’s decision (6). Three children did not complete the follow-up (T2) questionnaires after the NF. The 91 subjects who completed the treatment were similar to the 39 who dropped out of the treatment regarding their socio-demographic status, in terms of their family structure, number of siblings, parents’ education, economic resources, and other support. There were no significant differences in their academic skills (IQ mean difference: -9.8, 95 % CI: (-7.9, 5.9)).

### Results within the treatment groups

Parents reported significant changes in all scales within the three treatment groups (unadjusted, p < 0.001, Table 2). The change was quite strong for hyperactivity, but weak for attention. Consequently, the size of the change for the total scale was dominated by the hyperactivity change. After adjusting for age and sex, no significant changes were found (adjusted, Table 2).

**Table 2:** Parents report of ADHD core symptoms (attention, hyperactivity, total score) regarding the treatments (NF, MED, combined)

	**Pre treatment**			**Post treatment**			**Pre-Post change (T**_1_-T_2_)^**1**^						**Treatment effect**^**2**^	
**Barkley rating scores**	**N**	**Mean**	**95 % CI**	**N**	**Mean**	**95 % CI**	**N**	**Mean**^**3**^	**95 % CI**	**Effect size**^**4**^	**p-unadj.**^**5**^	**p-adj.**^**6**^	**p-uadj.**^**5**^	**p-adj.**^**6**^
Total											< .001	0,310	0.173	0,228
Medication	29	34,5	(30.6, 38.5)	22	27,8	(24.0, 31.6)	22	7.9	(4.5, 11.4)					
Neurofeedback + Medication	24	32,6	(28.6, 36.6)	22	23,7	(19.8, 27.6)	22	8,6	(5.0, 12.2)	1.76				
Neurofeedback	23	37	(33.9, 40.0)	19	26,7	(23.4, 30.1)	19	10,7	(7.6, 13.8)	2.25				
Attention											< .001	0,738	0,098	0,139
Medication	29	15,9	(13.8, 18.0)	22	15,2	(13.2, 17.2)	22	1,5	(-0.3, 3.3)	0.70				
Neurofeedback + Medication	24	15,9	(13.9, 18.0)	22	14,5	(12.0, 17.2)	22	1,1	(-0.7, 3.0)	0.46				
Neurofeedback	23	19,2	(17.2, 21.1)	19	16,6	(14.5, 18.7)	19	3,1	(1.6, 4.5)	2.08				
Hyperactivity											< .001	0,077	0,101	0,186
Medication	29	18,7	(16.3, 21.0)	22	12,5	(10.2, 14.4)	22	6,5	(4.3, 8.6)	2.45				
Neurofeedback + Medication	24	16,7	(14.3, 19.0)	22	9,1	(6.8, 11.4)	22	7,5	(4.9, 10.0)	1,75				
Neurofeedback	23	17,8	(15.9, 19.6)	19	10,1	(8.0, 12.2)	19	7,6	(5.6, 9.6)	2.88				

^1^ Tests for within-subject-contrast in the General linear modell (GLM). ^2^ Between-groups-effect in the GLM. ^3^ Mean of the differences, i.e. excluding drop outs at one missing point. ^4^ Effect size measure δ_RM_ (ref Morris). ^5^ Unadjusted model. ^6^ Model adjusted for age and sex.

### Results between the treatment groups

Based on the parents’ reports, we did not observe significant differences between the treatment groups. Neither the between-groups effects in the GLM nor the post-hoc tests showed any significant change scores, for any scales or pair of treatment groups (Table 2). We did not report the ESs or the results of the post-hoc tests since there were no significant differences.

Generally, our comparisons of the treatments had low powers. Although not significant, the NF group showed more than double the pre–post change in attention compared with the other two treatments (3.1 vs. 1.1 and 1.5 for the means). Additionally, we noted that the MED and NF + MED group had a confidence interval that included 0. The NF group had the largest ES δ_RM_ for all scales, but one should be careful interpreting this observation since those three scales are highly correlated. For all scales, we noted that the p-value for the unadjusted models were lower than for the adjusted models.

## Discussion

In the present controlled and randomized clinical trial, NF treatment seemed to improve the core symptoms of ADHD, as assessed by parental reports. In addition, NF and MPH produced similar improvements.

The strength of the present study was the effective randomization by age, sex, and intelligence, and the distribution of ADHD core symptoms (hyperactivity and attention). However, the number of subjects in each treatment group was somewhat low.

NF improved attention and hyperactivity symptoms in children and adolescents with ADHD. This is in accordance to findings in other studies, including a study by Kaiser, Thomsen and Othmer that found significant improvements in ADHD symptoms with NF for more than three in four ADHD subjects [32,44]. We did not find significant difference between the three investigated treatments in the improvement of ADHD core symptoms. This is in accordance with the work of Rossiter and Fuchs, who found in a rather large sample size that effects of NF on hyperactivity and attention ( ES 1.01-1.71) are equivalent to those obtained with stimulant drugs (0.80-1.80) [17,30]. Consequently, NF can be suggested to produce equivalent beneficial effects for ADHD as medications. Furthermore, our findings support the suggestion by Fuchs and Lubar to introduce NF as a treatment option for children with ADHD whose parents favored a non-pharmacological treatment [21,30].

However, regarding the improvement of the core symptoms of ADHD, nonspecific factors may contribute to the positive effects induced by NF [14,16]. Mainly, there are three nonspecific factors described in previous studies that may result in ADHD symptom improvement. These include the extraordinary amount of time spent with the therapist during NF, better motivation for changes in ADHD symptoms, and cognitive-behavioral training introduced under NF [26,32]. These factors may explain some improvement of hyperactivity, but may be a minor factor.

Even if we did not find significant differences in the core symptoms of ADHD, we observed a lower ES for the combined treatment for all symptoms. Such a tendency would lie in contrast to previous studies, which found NF and MPH was associated with the best improvements in the core symptoms of ADHD [11].

The results of the present study support the use of NF as an alternative treatment for ADHD, especially in the 20% of children with ADHD who do not respond to medications. In addition, findings from this study support previous suggestions that medications may be reduced when given in combination with other treatments for ADHD [21].

## Conclusions

NF produced a significant improvement in the core symptoms of ADHD, which was equivalent to the effects produced by MPH, based on parental reports. This supports the use of NF as an alternative therapy for children and adolescents with ADHD.

### Key messages

1. NF improves the core symptoms of ADHD based on parental reports.

1. NF and MPH produce equivalent improvements in the core symptoms of ADHD based on parental reports.

## Abbreviations

ADHD = Attention-deficit/hyperactivity disorder; NF = Neurofeedback; MED = Medication.

## Competing interests

The authors declare no potential conflicts of interests with regard to the authorship or publication of this article.

## Authors’ contributions

NSD and IBE made substantial contributions to the design, analysis, and interpretation of the data, and they were involved in drafting the manuscript and revising it critically. JA contributed to the statistical analyses and data interpretation. DG acquired the funding and contributed to the study design. All authors read and approved the final manuscript.

## Pre-publication history

The pre-publication history for this paper can be accessed here:

http://www.biomedcentral.com/1471-244X/12/107/prepub
